# Complete mitochondrial genome of *Cicindela* (*Cicindela*) *transbaicalica* (Coleoptera, Cicindelidae, Cicindelini)

**DOI:** 10.1128/mra.01519-25

**Published:** 2026-03-25

**Authors:** Guoqing Li, Hongbin Liang, Xun Bian, Bin Zhang

**Affiliations:** 1 College of Life Sciences and Technology, Inner Mongolia Normal University 71203 , Hohhot, China; 2 Key Laboratory of Zoological Systematics and Evolution, Institute of Zoology, Chinese Academy of Sciences 53024 , Beijing, China; 3 Guangxi Key Laboratory of Rare and Endangered Animal Ecology, Guangxi Normal University 12388, Guilin, China; 4 Biodiversity Conservation and Sustainable Utilization in Mongolian Plateau for College and University of Inner Mongolia Autonomous Region 71203 , Hohhot, China; University of Maryland School of Medicine, Baltimore, Maryland, USA

**Keywords:** mitogenome, *Cicindela* (*Cicindela*)* transbaicalica*, Cicindelini

## Abstract

We report the mitochondrial genome of *Cicindela* (*Cicindela*) *transbaicalica* Motschulsky, 1844, based on a specimen from Hulunbuir, Inner Mongolia, China. The 16,505 bp genome contains 13 protein-coding, 22 transfer RNA, and 2 ribosomal RNA genes, with gene content and arrangement typical of *Cicindela* species.

## ANNOUNCEMENT

**Fig 1:**
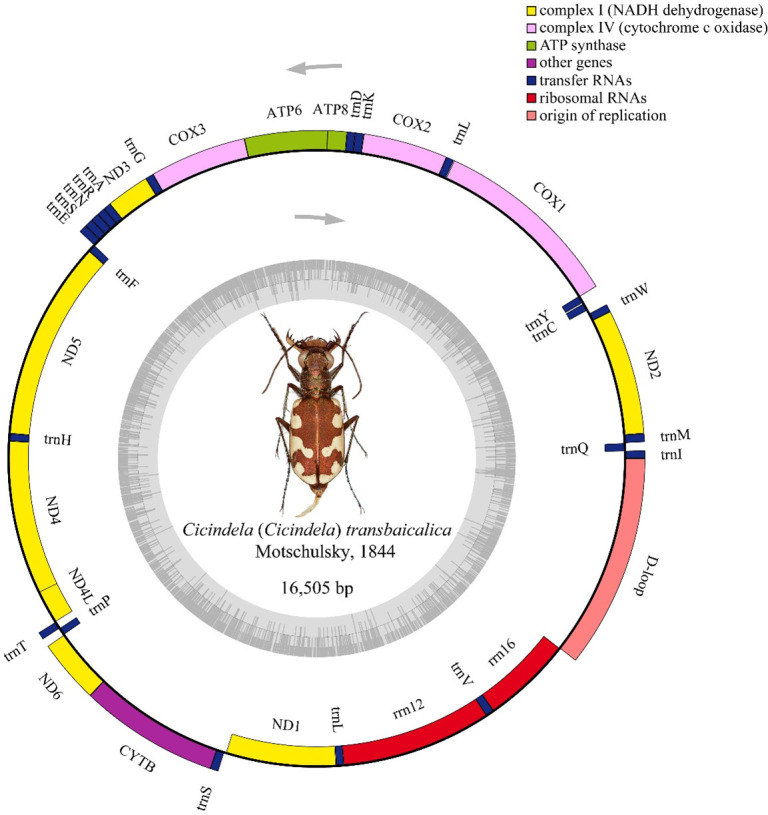
Circular map of the mitochondrial genome of *Cicindela* (*Cicindela*) *transbaicalica*. The center shows a specimen photo; the inner gray ring indicates GC content, and the outer double ring shows genes with arrows representing their transcription direction (forward on the outer ring and reverse on the inner ring).

*Cicindela* is among the most speciose genera within the family Cicindelidae and has a worldwide distribution (1). *Cicindela* (*Cicindela*) *transbaicalica* Motschulsky, 1844, is widely distributed in China, North Korea, South Korea, Japan, Mongolia, and Russia (2–4) and can be distinguished by a distinct metallic sheen on the elytra and by retaining the complete tri-maculate pattern, with the median hook-shaped band slightly constricted medially (5) (Fig. 1). Despite its broad distribution and clear morphology, molecular data remain limited, with partial molecular markers (e.g., COX1 and 28S ribosomal RNA [rRNA]) available. In this study, we sequenced and annotated the complete mitochondrial genome of *Cicindela* (*Cicindela*) *transbaicalica*.

The specimen of *Cicindela* (*Cicindela*) *transbaicalica* was collected in New Barag Left Banner, Hulunbuir, Inner Mongolia, China (49.170145°N, 118.408241°E; 615 m). The voucher specimen (IOZ(E)2610690) is deposited at the Institute of Zoology, Chinese Academy of Sciences. Total genomic DNA was isolated from hind femur tissue using the TIANamp Genomic DNA Kit (TIANGEN), following the manufacturer’s protocol. Approximately 0.2 μg of DNA was fragmented to ~350 bp using a Covaris LE220R-plus (Covaris, USA). Libraries were prepared using the Rapid Plus DNA Lib Prep Kit for Illumina (ABclonal, China) and sequenced on the DNBSEQ-T7 platform by Novogene (Beijing, China). Paired-end sequencing (2 × 150 bp) generated 35,851,488 raw reads. Raw reads were filtered using fastp v0.20.0 (6) to remove adapters, reads with Phred quality scores <5, and reads containing >10% ambiguous bases (N). The sequenced mitogenome data were assembled by NOVOPlasty 4.3.5 (7), yielding an N50 of 16,505 bp and GC content of 25.3%, and 649× average coverage. Based on BLAST results (8) and with reference to established phylogenies (9), the mitochondrial genome of *Cicindela puritana* (GenBank: MW442537) was selected as the reference sequence. For subsequent annotation, the start position was aligned to the reference sequence using Geneious Prime 2025.0.2 (https://www.geneious.com) (10). Gene boundaries were determined by comparison with the complete mitogenomes of two congeneric species from GenBank (*Cicindela aurulenta*: PX240909 and *Cicindela chinensis*: PV699634), and all initiation and termination codons were verified using the invertebrate mitochondrial genetic code (11, 12). Following annotation, nucleotide identities against related species were assessed via BLAST under default parameters. The graphical map of the assembled mitogenome was produced using the online tool OGDRAW (13).

The complete mitochondrial genome of *Cicindela* (*Cicindela*) *transbaicalica* is a circular molecule of 16,505 bp, comprising the typical set of 37 genes, including 13 protein-coding genes (PCGs), 22 transfer RNA (tRNA) genes, and 2 rRNA genes, along with a major noncoding control region (Fig. 1). Among the 13 protein-coding genes, except ND3 and ND1, which use TTG as the initiation codon, the other 11 PCGs start with the conventional ATN codons. The ND1 and ND3 usage of TTG as an atypical initiation codon has been reported in invertebrates, including Coleoptera (14, 15). Most PCGs terminate with complete stop codons (TAA/TAG), whereas COX2, ND5, and ND4 use incomplete termination codons (TA/T; Table 1). Incomplete termination codons (TA/T) found in several PCGs are presumed to be completed by post-transcriptional polyadenylation (16), as reported for other coleopteran mitogenomes (17, 18).

**TABLE 1:** Gene composition and annotation of *Cicindela* (*Cicindela*) *transbaicalica* Motschulsky, 1844

Gene	Type	Minimum nucleotide position	Maximum nucleotide position	Length	Start codon	Stop codon	Direction
tRNA-Ile	tRNA	1	64	64	–^*a*^	–	Forward
tRNA-Gln	tRNA	62	130	69	–	–	Reverse
tRNA-Met	tRNA	132	200	69	–	–	Forward
ND2	CDS	201	1220	1,020	ATA	TAA	Forward
tRNA-Trp	tRNA	1219	1284	66	–	–	Forward
tRNA-Cys	tRNA	1317	1383	67	–	–	Reverse
tRNA-Tyr	tRNA	1392	1457	66	–	–	Reverse
COX1	CDS	1450	2991	1,542	ATT	TAA	Forward
tRNA-Leu	tRNA	2995	3058	64	–	–	Forward
COX2	CDS	3059	3747	689	ATA	TA	Forward
tRNA-Lys	tRNA	3748	3817	70	–	–	Forward
tRNA-Asp	tRNA	3818	3882	65	–	–	Forward
ATP8	CDS	3883	4041	159	ATC	TAG	Forward
ATP6	CDS	4038	4712	675	ATA	TAA	Forward
COX3	CDS	4712	5500	789	ATG	TAA	Forward
tRNA-Gly	tRNA	5500	5565	66	–	–	Forward
ND3	CDS	5566	5919	354	TTG	TAG	Forward
tRNA-Ala	tRNA	5918	5981	64	–	–	Forward
tRNA-Arg	tRNA	5982	6045	64	–	–	Forward
tRNA-Asn	tRNA	6046	6112	67	–	–	Forward
tRNA-Ser	tRNA	6113	6179	67	–	–	Forward
tRNA-Glu	tRNA	6180	6246	67	–	–	Forward
tRNA-Phe	tRNA	6245	6310	66	–	–	Reverse
ND5	CDS	6311	8039	1,729	ATT	T	Reverse
tRNA-His	tRNA	8040	8103	64	–	–	Reverse
ND4	CDS	8104	9436	1,333	ATG	T	Reverse
ND4L	CDS	9430	9720	291	ATT	TAA	Reverse
tRNA-Thr	tRNA	9723	9786	64	–	–	Forward
tRNA-Pro	tRNA	9787	9852	66	–	–	Reverse
ND6	CDS	9854	10375	522	ATT	TAA	Forward
CYTB	CDS	10375	11511	1,137	ATG	TAG	Forward
tRNA-Ser	tRNA	11510	11577	68	–	–	Forward
ND1	CDS	11594	12544	951	TTG	TAG	Reverse
tRNA-Leu	tRNA	12546	12609	64	–	–	Reverse
16S RNA	rRNA	12610	13911	1,302	–	–	Reverse
tRNA-Val	tRNA	13912	13982	71	–	–	Reverse
12S RNA	rRNA	13983	14761	779	–	–	Reverse
CR	D-loop	14762	16505	1,744	–	–	Forward

^*a*^ –, not applicable.

## Data Availability

The complete mitochondrial genome sequence of *Cicindela* (*Cicindela*) *transbaicalica* is available in GenBank under accession number PX574207. The associated BioProject, BioSample, and SRA numbers are PRJNA1364839, SAMN53262588, and SRR36095933, respectively.
